# Grading in Gerodontology: A Framework

**DOI:** 10.1111/ger.70051

**Published:** 2026-05-17

**Authors:** Falk Schwendicke, Gerry McKenna, Frauke Müller, Azize Bell, Najla Chebib, Cristiane Da Mata, Helena Dujic, Jennifer Gallagher, Barbara Janssens, Danielle M. Layton, Manabu Kanazawa, Anastassia Kossioni, Sabrina Maniewicz, Koichiro Matsuo, Pedro Molinero Mourelle, Annika Neri, Takahiro Ono, Sungkrit Pojmonpiti, Foteini Spyraki, Kittipit Srisanoi, Saif S. Syed, Sayaka Tada, Georgios Tsakos, Terry R. Walton, W. Murray Thomson, Jean‐Philippe Haesler, Sophie Dartevelle, Murali Srinivasan

**Affiliations:** ^1^ Department of Conservative Dentistry, Periodontology and Digital Dentistry, LMU University Hospital LMU Munich Munich Germany; ^2^ Centre for Public Health Queen's University Belfast Belfast UK; ^3^ Center of Excellence in Precision Medicine and Digital Health, International Program in Geriatric Dentistry and Special Patients Care, Faculty of Dentistry Chulalongkorn University Bangkok Thailand; ^4^ Division of Gerodontology and Removable Prosthodontics, University Clinics of Dental Medicine University of Geneva Geneva Switzerland; ^5^ Poliklinik für Zahnärztliche Prothetik und Werkstoffkunde Universitätsmedizin der Johannes Gutenberg‐Universität Mainz Mainz Germany; ^6^ Restorative Dentistry Cork University Dental School and Hospital, UCC Cork Ireland; ^7^ Faculty of Dentistry, Oral & Craniofacial Sciences King's College London London UK; ^8^ ELOHA (Equal Lifelong Oral Health for All) Research Group, Department of Oral Health Sciences, Faculty of Medicine and Health Sciences Ghent University Ghent Belgium; ^9^ Department of Oral Health Ghent University Hospital Ghent Belgium; ^10^ School of Dentistry, Faculty of Health and Behavioural Sciences University of Queensland, & Specialist Private Practice St Lucia Queensland Australia; ^11^ Gerodontology and Oral Rehabilitation, Graduate School of Medical and Dental Sciences Institute of Science Tokyo Tokyo Japan; ^12^ Discipline of Gerodontology, Department of Prosthodontics, Dental School National and Kapodistrian University of Athens Athens Greece; ^13^ Department of Oral Health Sciences for Community Welfare, Graduate School of Medical and Dental Sciences Institute of Science Tokyo Tokyo Japan; ^14^ Department of Reconstructive Dentistry and Gerodontology, School of Dental Medicine University of Bern Bern Switzerland; ^15^ Department of Conservative Dentistry and Orofacial Prosthodontics, Faculty of Dentistry Complutense University of Madrid Madrid Spain; ^16^ Ramon y Cajal Research Institute (IRYCIS) Complutense University of Madrid Madrid Spain; ^17^ Department of Geriatric Dentistry Osaka Dental University Osaka Japan; ^18^ Department of Neurology Ghent University Hospital Ghent Belgium; ^19^ Dental Department Chulabhorn Hospital, Chulabhorn Royal Academy Bangkok Thailand; ^20^ Clinic of General‐, Special Care‐ and Geriatric Dentistry, Center for Dental Medicine University of Zurich Zurich Switzerland; ^21^ Faculty of Dentistry Khon Kaen University Khon Kaen Thailand; ^22^ Department of Epidemiology and Public Health University College London London UK; ^23^ Institute of Dentistry Queen Mary University of London London UK; ^24^ Faculty of Dentistry National University of Singapore Singapore Singapore; ^25^ Department of Epidemiology and Public Health University College London (UCL) London UK; ^26^ School of Dentistry, Faculty of Medical Sciences University of Sydney, & Specialist Private Practice Sydney New South Wales Australia; ^27^ Faculty of Dentistry University of Otago Dunedin New Zealand; ^28^ Swiss Dental Association SSO Bern Switzerland; ^29^ The European Regional Organization of the FDI World Dental Federation ERO‐FDI Gümligen Switzerland

**Keywords:** case complexity, frailty, geriatric dentistry, hyposalivation, multimorbidity, nutrition, older adults, socio‐economic status, treatment planning, xerostomia

## Abstract

**Introduction:**

Understanding and anticipating oral health deterioration in older adults requires more than describing their current oral and general health. Risk factors arising from oral, systemic and social domains strongly influence disease progression, care complexity and the ability to maintain function over time. We aimed to establish a framework for staging and grading oral health and disease in older adults, with grading systematically assessing and describing determinants of oral health deterioration. The developed framework is intended to support care planning, education, research and health‐service policy.

**Methods:**

Eight reviews examining oral, systemic and social risk factors for worsening oral health in older adults informed framework development. Additional existing reviews were also considered. A 2.5‐day international workshop involving 31 experts from 13 countries was conducted, during which structured subgroup discussions and Delphi‐style anonymous voting were used to refine and agree on the framework. Consensus was predefined at 75% agreement, and that was subsequently reached for all statements in a single voting round. The current paper presents the grading aspect of the developed framework; it should be used jointly with the paper on staging oral health in older adults.

**Results:**

Grading encompasses three levels of risk factors: (1) oral‐level factors such as dental caries risk, periodontitis risk and risks associated with the maintenance of existing dental prostheses; (2) systemic‐level factors including xerostomia, multimorbidity, polypharmacy and nutritional aspects and (3) social‐level factors such as social status and social support. Together, these domains reflect the likelihood of oral health deterioration, the intensity and frequency of required supportive care and the potential complexity of clinical management. Grading complements staging—which describes the current oral health status—by identifying the underlying risks shaping each older adult's oral health trajectory.

**Conclusion:**

Grading provides a structured, evidence‐based method for assessing risk and care complexity in older adults. When combined with staging, it should enable clinicians, caregivers and policymakers to classify cases comprehensively, tailor preventive and supportive interventions, and inform resource planning, surveillance, research and education. Future work should focus on validating, disseminating and implementing the framework across diverse care settings.

## 1 Introduction

Oral health in older adults is determined not only by present conditions but also by the interplay of systemic, functional, social, educational and economic factors that shape how oral health evolves over time. Although the clinical status of teeth, periodontium or mucosa describes the *current* situation, this information alone does not capture the likelihood of deterioration or the level of support required to maintain oral function and quality of life. Ageing is accompanied by functional impairment and psychosocial changes and often also by multimorbidity, polypharmacy, nutritional vulnerability, all of which affect oral health outcomes.

Existing diagnostic or screening tools in gerodontology largely focus on identifying disease or treatment need but rarely address *risk* or *care complexity*. They seldom integrate systemic and social determinants of oral health or the patient's ability to engage in preventive and supportive care. These flaws limit the predictive value of current approaches and hinder targeted, resource‐efficient management.

The current work aimed to develop and achieve consensus on a framework to enable comprehensive assessment of older adults' oral health—capturing their general and oral function and disease status through *staging*, and identifying oral, systemic, and socio‐economic/social risk factors influencing care complexity, capability, needs and management through *grading*. Grading extends the framework from ‘what is’ to ‘what may happen’. It links oral conditions with broader determinants of health that collectively determine resilience or vulnerability. A complementary paper details the staging dimension (to be added); the current paper describes in full the rationale, methods and outcomes of the grading framework, which is intended to provide a foundation for preventive and personalised oral health care for older and very old adults.

## 2 Methods

### 2.1 Process

A four‐member steering committee (FS, MS, GM and FM) led the development of the grading component within the broader international project on staging and grading in gerodontology. Building upon an initial scoping exercise and conceptual workshop in Munich (in December 2024), the committee defined the specific aims and domains for grading, emphasising factors that predict deterioration and influence care complexity rather than current disease presence.

The process was structured in three main phases: (1) *evidence synthesis*, drawing on multiple systematic reviews; these have been outlined in the publication on staging (to be added); (2) *expert consensus*, through an international workshop and Delphi‐style voting and (3) *framework consolidation* and refinement by the steering group. All steps were conducted in accordance with the Guidance on Conducting and Reporting Delphi Studies (CREDES) (Jünger et al. [1]).

### 2.2 Aspects

Grading was designed to complement staging by addressing oral, systemic and social determinants that modulate risk and prognosis. The steering group identified key domains based on systematic reviews covering:

- caries and periodontal disease progression and management complexity;
- prosthetic maintenance requirements;
- xerostomia, salivary hypofunction, and their implications for oral health and function;
- multimorbidity, polypharmacy and frailty;
- nutritional status and its bidirectional relationship with oral function; and
- social status and social support as mediators of self‐care and access to treatment.

The reviews were conducted according to PRISMA guidelines. Their findings informed the formulation of preliminary grading domains and parameters which were subsequently refined through the consensus process.

### 2.3 Consensus Conference and Voting

The international consensus conference took place in Geneva (Switzerland) from 26 to 28 August 2025. Thirty‐one experts from 13 countries participated, representing clinical gerodontology, prosthodontics, periodontology, nutrition, public health, epidemiology and geriatric medicine. Stakeholders from professional organisations (ECG, IADR, FDI, IADH, EuGMS and SSO) were also involved.

Following presentations of the systematic reviews and the preliminary framework, three thematic subgroups were formed to discuss and refine statements pertaining to:

1. *oral‐level* risk factors (e.g., caries risk, periodontal risk and prosthetic maintenance);
2. *systemic‐level* risk factors (e.g., xerostomia, multimorbidity and nutrition); and
3. *social‐level* determinants (e.g., social status, social support).

Each subgroup proposed grading criteria categorised as Grades A–C (low, moderate and high risk). The proposals were presented and debated in plenary sessions before anonymous voting.

Voting used Mentimeter (Stockholm, Sweden). Participants could vote *agree*, *disagree* or *abstain*, and comments were recorded. Consensus was predefined as ≥ 75% affirmative responses among votes cast. Two voting rounds were planned, but all statements achieved consensus in the first round. Further details on the involved methods are provided elsewhere (to be added).

## 3 Results

**FIGURE 1:**
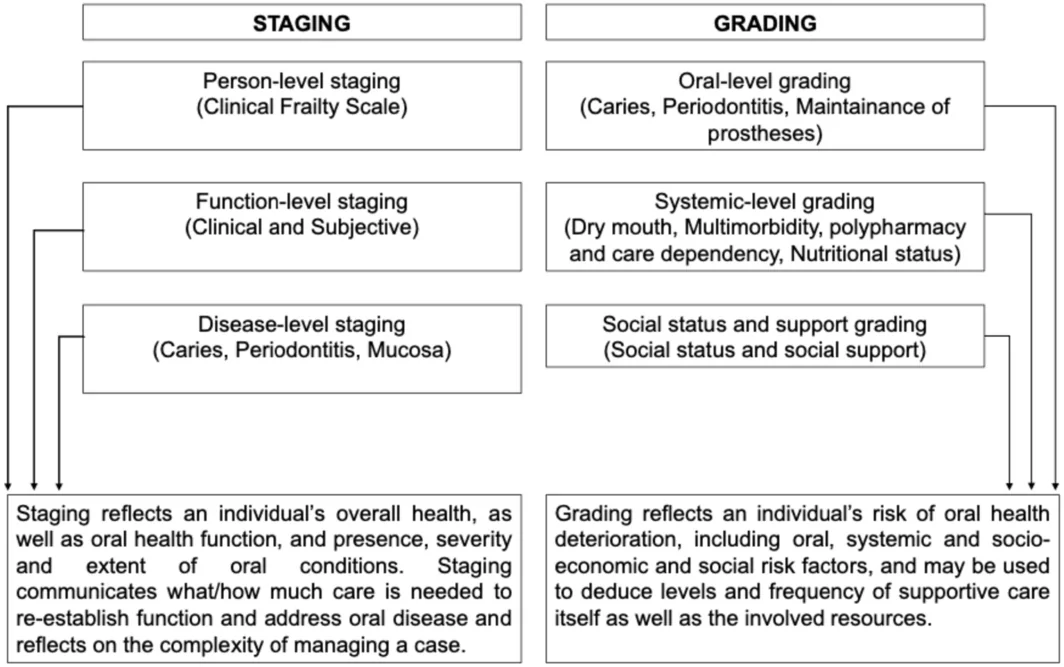
Flowchart guiding staging and grading. Copyright and cross‐referencing to be sorted by Wiley.

Staging and grading were assumed to capture an individual's oral health status and function, risk factors for rapid oral health deterioration and complexity of management needs, whether applicable for education and research, or for surveillance purposes, and to complement health services and policy development. Figure 1 and Table 1 summarise the resulting staging and grading framework.

**TABLE 1:** Aspects to consider during staging and grading.

Domain	Stage (I–III)	Grade (A–C)
Person‐level	Frailty: CFS 1–3 = Stage I; CFS 4–6 = Stage II; CFS 7–9 = Stage III	—
Function‐level (clinical)	Stage I = shortened dental arch; Stage II = less than shortened arch but bilateral occlusal support; Stage III = no bilateral occlusal support	—
Function‐level (subjective)	Stage I = no oral impact; Stage II = one oral impact; Stage III = > 1 oral impact/no answer	—
Caries	Stage I = no active lesions; Stage II = active lesions w/o pulpal involvement; Stage III = active lesions w/pulpal involvement/infection	Grade A = no active caries/no risk factors; Grade B = no active caries but risk factors; Grade C = active caries or high risk
Periodontitis	Stage I = PPD ≤ 4 mm, no mobility II/III; Stage II = PPD > 4 mm, no mobility II/III; Stage III = PPD > 4 mm + mobility II/III	Grade A = minimal progression (%BL/age < 0.25, no bone loss in 5 years); Grade B = moderate progression (%BL/age 0.25–1); Grade C = rapid progression (%BL/age > 1, ≥ 2 mm loss in 5 years)
Mucosa	Stage I = healthy; Stage II = non‐severe lesion (e.g., candidiasis, pressure ulcer); Stage III = severe/potentially malignant lesion, incl. MRONJ, etc.	—
Prostheses	—	Grade A = no prosthesis; Grade B = prosthesis well‐designed, manageable; Grade C = prosthesis defective, poorly fitting, or not manageable for patient
Dry mouth	—	Grade A = no/mild dry mouth (normal flow, negative mirror test); Grade B = moderate xerostomia or SGH; Grade C = severe xerostomia or SGH (mirror sticks, very low flow)
Multimorbidity/polypharmacy/dependency	—	Grade A = < 5 meds, no xerogenic drugs, CFS 1–3, no major geriatric conditions; Grade B = 5–9 meds or xerogenic drugs or CFS 4–6; Grade C = ≥ 10 meds or CFS 7–9 or major geriatric conditions
Nutrition	—	Grade A = normal MNA ≥ 24/MNA‐SF ≥ 12, no oral limitations; Grade B = at risk (MNA 17–23.5/MNA‐SF 8–11) or oral limitations; Grade C = malnourished (MNA < 17/MNA‐SF ≤ 7, calf circ. < 31 cm)
Social status	—	Grade A = high status (MacArthur 8–10, high income, tertiary education); Grade B = medium (MacArthur 3–7, medium income, secondary education); Grade C = low (MacArthur 0–2, low income, primary or lower education)
Social support	—	Grade A = not living alone; Grade B = living alone with support; Grade C = living alone without support

*Note:* Copyright and cross‐referencing to as above be sorted by Wiley.

Abbreviations: BL, bone loss; CFS, Clinical Frailty Scale; MNA, Mini Nutritional Assessment; MRONJ, medication‐related osteonecrosis of the jaw; PPD, probing pocket depth; SGH, salivary gland hypofunction.

As outlined in the publication on staging, the framework's application is not limited to specific age groups, since the older adult group has varying definitions, and relevant aspects (including frailty) may also apply to younger groups. Notably, the majority of the reviews supporting the grading process set an age threshold of 75 years or above to describe the target group.

Grading assesses oral‐level, systemic‐level and social‐level aspects. The agreement of the group (yes votes per all votes or abstentions) for each item is indicated below.

### 3.1 Oral‐Level Grading

#### 3.1.1 Caries Grading (Agreement: 28/30)

Caries grading reflects the likelihood of future development or progression of dental caries in older adults. This is particularly important in gerodontology, where root surface caries and recurrent lesions around existing restorations and prostheses are commonly encountered.

Caries risk is influenced by multiple local and behavioural factors. Caries risk assessment systems, such as *CAMBRA* or *Cariogram*, have been applied and validated in older populations for both coronal and root surface caries [2, 3]. A key indicator remains *past caries experience* (e.g., DMFT, DMFS including root surfaces), because it is a strong predictor of future disease experience.

Risk factors considered in grading were

- *Visible plaque* **—**indicating insufficient oral hygiene and a high bacterial load.
- *Exposed root surfaces/loss of papillae* **—**increasing susceptibility to root surface caries and food impaction.
- *Inadequate fluoride use* **—**brushing less frequently than twice daily with 1450 ppm fluoride toothpaste (acknowledging that fluoride concentrations vary among markets and regulatory frameworks) reduces caries protection.
- *Frequent consumption of fermentable carbohydrates* **—**regular consumption of sugary drinks or snacks drives cariogenic activity and sustained demineralization of dental tissues.
- *Presence of dental prostheses*—both fixed and removable dental prostheses can create plaque‐retentive niches, and teeth adjacent to crowns, bridges or implants are at higher risk due to difficulties in cleaning.
- *Dry mouth (xerostomia)* **—**usually medication‐induced, this elevates caries risk by reducing salivary buffering and triggering the seeking of relief with sugary drinks.

**Table:** 

Grade	Criteria
Grade A (low risk)	– One of the validated caries risk assessment systems indicates low risk or – Neither active caries nor risk factors
Grade B (moderate risk)	– One of the validated caries risk assessment systems indicates moderate risk or – No active caries but risk factors
Grade C (high risk)	– One of the validated caries risk assessment systems indicates high risk or – Active caries with or without risk factors

Guideline for application

- *Scope of use*: Caries grading complements caries staging by indicating the *future risk trajectory* for disease, supporting individualised preventive and therapeutic planning.
- *Procedure*: Use a validated caries risk assessment tool where available. Where this is not feasible, apply the defined risk factors to assign a grade (A–C).
- *Practical note*: In mixed presentations (e.g., no active lesions but multiple strong risk factors), assign the higher risk grade to ensure adequate preventive or supportive care.

#### 3.1.2 Periodontitis Grading (Agreement: 24/30)

Periodontitis grading reflects the likelihood of future disease progression. Although staging describes the current severity of periodontal breakdown, grading incorporates longitudinal changes as well as risk factors or indicators. In older adults, periodontitis risk is particularly shaped by age‐related immune alterations, cumulative exposure to plaque and the coexistence of systemic conditions. Validated concepts from the 2017 EFP/AAP Classification of Periodontal and Peri‐Implant Diseases and Conditions [4] are applied here, adapted for the gerodontological context.

**Table:** 

Grade	Criteria
Grade A (low risk)	– No periodontal bone loss over previous 5 years or – % BL/age < 0.25% or – Heavy biofilm but low levels of periodontal destruction
Grade B (moderate risk)	– < 2 mm periodontal bone loss over previous 5 years or – % BL/age 0.25%–1% or – Destruction is proportionate to biofilm
Grade C (high risk)	– ≥ 2 mm periodontal bone loss over previous 5 years or – % BL/age > 1% or – Limited amount of biofilm but high periodontal destruction

Abbreviation: BL, bone loss.

Guideline for application

- *Scope of use*: Periodontitis grading should be applied in conjunction with periodontal staging to indicate risk of future breakdown, guide supportive therapy intervals, and determine the intensity of preventive and therapeutic interventions.
- *Procedure*: Assess past radiographs and clinical records (if available) to evaluate progression over the last 5 years. Calculate % bone loss relative to age as an alternative indicator, as follows: First, measure the distance from the cemento‐enamel junction to the alveolar crest to determine mesial and distal bone loss, and then measure the distance from the cemento‐enamel junction to the root apex to obtain total root length, again mesial and distal. The proportion of mesial and distal bone loss is expressed as a percentage of the total root length and then divided by the patient's age to adjust for age‐related progression. If no radiographs are available, assess whether the extent and amount of biofilm is commensurate with periodontal destruction.

#### 3.1.3 Grading of Prosthesis Maintenance Needs (Agreement 28/30)

Grading of prosthesis maintenance needs reflects the extent to which existing dental prostheses—whether fixed or removable—pose ongoing requirements for upkeep, repair or professional support. In older adults, prostheses can compromise oral health by affecting function, plaque retention, aesthetics and comfort. Grading considers whether the maintenance demands of a prosthesis align with the individual's capabilities and whether the prosthesis is well designed and adapted. Poorly manufactured, ill‐fitting or defective prostheses increase maintenance needs, compromise oral health, and may accelerate disease progression and impair psycho‐social well‐being. Examples of poorly designed or defective prostheses include those with:

- inadequate aesthetics (e.g., unnatural appearance, poor integration with existing dentition);
- overhanging or ill‐fitting margins;
- porous or rough materials prone to plaque accumulation;
- insufficient retention or stability or poorly fitting intaglio surfaces;
- fractures or advanced wear; or
- poor cleansability (e.g., inaccessible areas around bridges or implant superstructures).

**Table:** 

Grade	Criteria
Grade A (low risk)	– No prosthesis
Grade B (moderate risk)	– Prosthesis with maintenance needs being in line with patient's capabilities and – No problems (well designed and manufactured, well adapted)
Grade C (high risk)	– Prosthesis with maintenance needs not in line with patient's capabilities or – Poorly designed, manufactured or ill‐fitting prosthesis or prosthesis with defects or wear

Guideline for application

- *Scope of use*: This grading should be applied to evaluate how prostheses contribute to long‐term care needs and potential risks in older adults.
- *Procedure*: Assess whether the patient has a prosthesis and, if so, evaluate its design, fit, material quality, wear and cleansability. Consider whether the required maintenance is realistically manageable for the individual. Assign Grades A–C accordingly.

### 3.2 Systemic‐Level Grading

#### 3.2.1 Dry Mouth Grading (Agreement 28/29)

Dry mouth grading captures the severity of xerostomia (subjective sensation of oral dryness) and salivary gland hypofunction (SGH, objectively measured as low salivary flow). Xerostomia affects about 1 in 5 older adults over the age of 50 years [5], whereas SGH is less common but clinically relevant, with pooled prevalences of 8% for unstimulated flow (≤ 0.1 mL/min) and 13% for stimulated flow (≤ 0.7 mL/min). Although distinct, xerostomia and SGH coincide in many people, and both contribute to higher risks of caries, mucosal lesions, impaired chewing and swallowing and compromised quality of life.

Risk factors include polypharmacy (especially xerogenic drugs such as antihypertensives, antidepressants and diuretics), head and neck radiation, autoimmune diseases (e.g., Sjögren's syndrome), dehydration and low fluid intake, along with some neurodegenerative conditions. Grading reflects the combined burden of symptoms and measured salivary function.

**Table:** 

Grade	Criteria
Grade A (low risk)	– SXI‐D score = 5 or SXI score = 5 or XI score = 11 reports no/mild xerostomia or – Single question response = ‘never’ or ‘occasionally’ or – Normal USFR (> 0.2 mL/min) or SSFR (> 1.0 mL/min) or – Mouth mirror/tongue depressor does not stick to the oral mucosa
Grade B (moderate risk)	– SXI‐D score = 6–10 or SXI score = 6–15 or XI score = 12–33 reports moderate xerostomia or – Single question response = ‘frequently’ or – USFR 0.1–0.2 mL/min or SSFR 0.7–1.0 mL/min
Grade C (high risk)	– SXI‐D score > 10 or SXI score > 15 or XI score > 33 reports severe xerostomia or – Single question response = “always” or – USFR ≤ 0.1 mL/min or SSFR ≤ 0.7 mL/min or – Mouth mirror/tongue depressor sticks to the oral mucosa

Abbreviations: SXI, summated xerostomia inventory; SXI‐D, modified German version of the xerostomia inventory questionnaire; USFR, unstimulated salivary flow rate; XI, xerostomia inventory.

Guideline for application

1. Use any of the tests to assess xerostomia and/or SGH.
2. Where feasible, use a validated xerostomia inventory (XI: 11 items, 5‐point Likert scale; SXI: 5 items, 5‐point Likert; SXI‐D: 5 items, 3‐point Likert) [6, 7].
3. Alternative, use a single‐item xerostomia question (‘How often does your mouth feel dry?’ Response options: ‘Never’, ‘Occasionally’, ‘Frequently’ and ‘Always’. The latter two response options indicate xerostomia).
4. Consider objective salivary flow measures (unstimulated or stimulated) if available.
5. If testing is not possible, undertake a tongue depressor or mouth mirror adherence test.
6. The most Severe Score on the above criteria determines the grade.

#### 3.2.2 Multimorbidity, Polypharmacy and Care Dependency Grading (Agreement 27/29)

Multimorbidity, polypharmacy and care dependency are highly prevalent in older adults and substantially influence oral health trajectories and the complexity of dental care. Multimorbidity, defined as the presence of two or more chronic conditions, affects up to 83% of older adults, whereas polypharmacy (≥ 5 medications) is present in about half of them. Both are associated with higher risks of caries, periodontal disease, tooth loss, xerostomia and impaired oral health‐related quality of life (OHRQoL).

Care dependency adds another dimension: it not only reflects current treatment capacity but also predicts further deterioration in oral health. For consistency, dependency is graded here using the *Clinical Frailty Scale* (*CFS*), which is already applied in person‐level staging. This integrated grading accounts for the cumulative systemic and functional burden that shapes oral care needs in older adults.

**Table:** 

Grade	Criteria
Grade A (low risk)	– < 5 medications and – No medications affecting oral health and – Not dependent (CFS: 1, 2 or 3); without a major geriatric condition that could impact oral health
Grade B (moderate risk)	– 5–9 medications or – Medications affecting oral health or – Mild‐to‐moderate dependency (CFS: 4, 5 or 6); without a major geriatric condition that could impact oral health
Grade C (high risk)	– 9 medications or – High dependency (CFS: 7, 8 or 9) or – Major geriatric conditions that could impact oral health

Abbreviation: CFS, Clinical Frailty Scale.

Guideline for application

1. *Multimorbidity*: Consider the presence of major geriatric conditions such as sarcopenia, diabetes, Parkinson's disease, cardiovascular conditions, osteoporosis, depression, cognitive impairment, malnutrition, dysphagia, incontinence and sensory impairments (hearing or vision) and among others.
2. *Polypharmacy*: Count all regularly‐taken therapeutic substances (including those in combination drugs, whether prescribed or over‐the‐counter medications). Identify whether any are known to affect oral health (e.g., xerogenic drugs).
3. *Care dependency*: Assess the individual's dependency using the *Clinical Frailty Scale* (*CFS*).
4. *Final grading*: The most severe score on the above criteria (MM, polypharmacy or dependency) determines the grade.

#### 3.2.3 Nutritional State Grading (Agreement 28/29)

Nutritional state is closely linked with oral health in older adults. Malnutrition and risk of malnutrition are particularly common among frail and institutionalised individuals, with impaired nutritional status being associated with poor oral health. Validated screening tools—such as the *Mini Nutritional Assessment* (*MNA*) and its short form (MNA‐SF)—are widely used to detect malnutrition or risk thereof [8]. Where self‐reporting is not possible, calf circumference can be used as a proxy measure. Obesity has not been incorporated into these guidelines and should be addressed in future research.

**Table:** 

Grade	Criteria
Grade A (low risk)	– Normal nutrition (MNA ≥ 24; MNA‐SF: 12–14; Calf circumference ≥ 31 cm) and^a^ – No reported oral limitations affecting food intake
Grade B (moderate risk)	– At risk of malnutrition (MNA: 17–23.5; MNA‐SF: 8–11) or – Normal nutrition (MNA ≥ 24; MNA‐SF: 12–14; Calf circumference ≥ 31 cm) and oral limitations present affecting food intake^a^
Grade C (high risk)	– Malnourished (MNA < 17; MNA‐SF 0–7; Calf circumference < 31 cm)^a^

Abbreviations: MNA, Mini Nutritional Assessment; MNA‐SF, Mini Nutritional Assessment‐Short Form.

^a^ The cutoff values for calf circumference should be adapted to ethnicity and obesity as appropriate.

Guideline for application

1. *Screening*: Use the *MNA* or *MNA‐SF* questionnaire.
  ○ MNA: ≥ 24 = normal; 17–23.5 = at risk; < 17 = malnutrition.
  ○ MNA‐SF: 12–14 = normal; 8–11 = at risk; 0–7 = malnutrition.
2. *Proxy measure*: If self‐reporting is not possible, measure calf circumference (cut‐off < 31 cm indicates malnutrition).
3. *Oral contribution*: Assess for oral limitations such as impaired chewing, swallowing difficulties, teeth with severe mobility, broken teeth, missing teeth or ill‐fitting dentures.
4. *Final grading*: The most severe score on the above criteria (nutritional assessment or oral limitation) determines the grade.

### 3.3 Social Status and Support

#### 3.3.1 Social Status (Agreement: 30/30)

Social status is a key determinant of oral health and oral health‐related quality of life in older adults. It influences health literacy, access to care, preventive behaviours and capacity to adhere to maintenance regimens. This grading incorporates three possible measures: subjective social status, household income/wealth or level of education.

- The MacArthur Scale of Subjective Social Status (10‐point ladder, self‐rated) is prioritised, as it has been validated across populations and captures perceived social position [9]. For this scale, respondents view a drawing of a ladder with 10 rungs, and read or hear that the ladder represents where people stand in society. Respondents further read or hear: ‘At the top of the ladder are the people who are the best off, those who have the most money, most education and best jobs. At the bottom are the people who are the worst off, those who have the least money, least education, worst jobs or no job. Please place an “X” on the rung that best represents where you think you stand on the ladder’.
- Where this is not feasible, household income/wealth or educational attainment can be used as alternatives. Educational categories should be adapted to local systems and cohort‐specific contexts (e.g., older generations may have followed different educational trajectories).
- Only one instrument should be applied per case, in order to ensure consistency.

**Table:** 

Grade	Criteria
Grade A (low risk)	– MacArthur Scale 8–10 or – Household income/wealth—high or – Education tertiary
Grade B (moderate risk)	– MacArthur Scale 3–7 or – Household income/wealth—medium or – Education secondary or
Grade C (high risk)	– MacArthur Scale 0 to 2 or – Household income/wealth—low or – Education primary or lower

Guideline for application

1. *Instrument selection*: Prioritise the MacArthur Scale of Subjective Social Status. If not available, use household income/wealth. If neither is available, use educational attainment. Apply only one instrument for each case.
2. *MacArthur Scale*: Ask the individual to place themselves on a 10‐step ladder, where 10 = highest social standing and 0 = lowest.
3. *Income/wealth*: Classify into high, medium or low based on local standards and cost of living adjustments.
4. *Education*: Categorise as primary or lower, secondary or tertiary. Adapt classification to local education systems and the cohort's age.
5. *Final grading*: Use the criteria in the table to assign Grades A–C, as appropriate.
6. *Missing data*: Assign Grade B by default, unless the individual is known to be on social allowance/assistance, in which case assign Grade C.

#### 3.3.2 Social Support Grading (Agreement: 25/29)

Social support plays a crucial role in determining older adults' ability to maintain oral health and access dental care. Support from family members, caregivers or community networks can compensate for functional or systemic limitations, whereas a lack of support can exacerbate vulnerability and lead to deterioration in oral health. This grading focuses on living arrangements and the presence or absence of effective social support, which strongly influence self‐care, adherence to dental maintenance, and the ability to seek professional help.

**Table:** 

Grade	Criteria
Grade A (low risk)	– Not living alone
Grade B (moderate risk)	– Living alone with social support when needed
Grade C (high risk)	– Living alone without social support

Guideline for application

1. *Living arrangement*: Determine whether or not the individual lives alone.
2. *Social support*: If living alone, determine whether regular, reliable social support would be available (e.g., from family, friends, caregivers or organised services) if needed.
3. *Final grading*: Assign Grades A–C according to the table.
4. *Note*: Support should be available when needed, *practical, and consistent* (e.g., help with daily living, oral hygiene or dental visits), not just occasional or nominal.

## 4 Discussion

The developed grading framework complements the descriptive staging system by integrating determinants of progression, maintenance difficulty and social vulnerability into a single, structured approach. Staging and grading represent complementary but distinct components of the framework. Staging describes the current oral and general health status of an older adult at a given point in time. It captures frailty at the person level, functional capacity at the clinical and subjective level and the presence and severity of key oral diseases. Staging therefore answers the question ‘Where is the patient now?’ and defines baseline treatment needs and feasibility of care. Grading, in contrast, contextualises staging by incorporating factors that modify risk, complexity and expected disease trajectory. It addresses the forward‐looking question ‘What factors influence how oral health may change in the future and how care can realistically be delivered?’ Grading may hence pave the way for personalised care and could allow operationalising risk‐based, value‐oriented care for ageing populations. Importantly, staging and grading are not aggregated into a single composite score. Instead, clinical reasoning is based on the pattern of stages and grades across domains. For example, identical disease‐stage profiles may require different management strategies when grading reveals differences in systemic burden, social support or care dependency. Conversely, high grading in the absence of advanced disease staging may trigger intensified preventive or supportive interventions. The interaction of staging and grading enables a structured yet flexible interpretation of patient cases and individualised clinical decision‐making, interdisciplinary communication and population‐level analyses without oversimplification. At this stage, the framework is intentionally designed for comprehensive and transparent assessment rather than score‐based classification.

At the oral health level, grading captures the risk of future caries and periodontitis incidence and/or increment, along with the functional resilience and maintenance needs of dental prostheses. These local factors remain modifiable targets for dental interventions. At the systemic level, xerostomia, multimorbidity and nutrition are identified as mediators linking oral and general health. Polypharmacy‐induced salivary dysfunction, for example, amplifies risk across multiple oral domains, whereas nutritional deficits exacerbate frailty and slow recovery from oral disease. The social level acknowledges the profound influence of social status and support on oral health behaviours and access to care.

Clinicians can use grading to stratify patients according to risk, thereby determining the required frequency of supportive care, preventive intensity and degree of interdisciplinary coordination. In dental education, the framework provides a structure to teach comprehensive geriatric assessment, bridging the gap between the biomedical and social determinants of health. In research, grading supports harmonised data collection and health services investigations. It enables exploration of how systemic and social risks interact with oral outcomes and how targeted interventions modify these relationships. For policymakers, the framework provides a potential basis for remuneration systems and service models that reflect patient complexity rather than procedural quantity.

The framework's main strength lies in its empirical foundation and international consensus. Its tripartite structure—oral, systemic and social—mirrors the multidimensional nature of ageing. Nonetheless, validation is required to confirm its validity and predictive accuracy across settings, as discussed elsewhere (to be added). Under‐representation of certain stakeholder groups in the developmental process—particularly caregivers and professionals from low‐ and middle‐income countries—is a limitation. Further testing in diverse health systems will be essential to ensure global applicability. Specifically, implementation and the potential need for adaptation in low and middle‐income settings should be assessed. For example, the MacArthur Scale of Subjective Social Status has been widely used across diverse cultural contexts; however, its validity depends on contextual interpretation. Future work will focus on developing abbreviated clinical tools for use by general dentists, hygienists and non‐dental caregivers; integrating the grading algorithm into electronic health records; and validating predictive performance in longitudinal studies. Investigating the interaction between staging and grading scores may allow the creation of a composite oral health complexity index suitable for clinical decision support and policy evaluation.

## 5 Conclusions

Together with the staging component, which describes an individual's current general health, oral function and disease status, the grading framework completes a comprehensive system for assessing oral health in older adults. Grading adds a forward‐looking perspective by classifying oral, systemic and social factors that influence risk and care complexity. In combination, staging and grading provide a coherent structure for defining clinical situations, anticipating deterioration, and guiding preventive, active and supportive care. The full framework offers a common language for clinical practice, education, research, surveillance and policy development. Future work should focus on validation and integration of both components into routine care pathways.

## Author Contributions

Each author made substantial contributions to the conception and design of the work and/or the acquisition, analysis or interpretation of data; participated in drafting the manuscript or revising it critically for important intellectual content; approved the final version to be published and agreed to be accountable for all aspects of the work.

## Funding

The consensus workshop was supported by the Gerodontology Association.

## Ethics Statement

The authors have nothing to report.

## Consent

All authors agreed to publishing.

## Conflicts of Interest

The authors declare no conflicts of interest.

## Data Availability

The authors have nothing to report.
